# Triphenylphosphonium (TPP)-Conjugated Quinolone Analogs Displayed Significantly Enhanced Fungicidal Activity Superior to Its Parent Molecule

**DOI:** 10.3390/jof9060685

**Published:** 2023-06-19

**Authors:** Jiayao Wang, Xuelian Liu, Fahong Yin, Yanjun Xu, Bin Fu, Jiaqi Li, Zhaohai Qin

**Affiliations:** 1 College of Science, China Agricultural University, Beijing 100193, China; ian_wangjiayao@163.com (J.W.); yinfahong@cau.edu.cn (F.Y.); xyj-323@163.com (Y.X.); fubinchem@cau.edu.cn (B.F.); jiaqili@cau.edu.cn (J.L.); 2 Pharmaceutical Institute, Xinjiang University, Urumqi 830046, China; lxl_nuo@126.com

**Keywords:** mitochondria-targeted, quinolone analogs, fungicidal activity

## Abstract

Although 1-hydroxy-4-quinolone derivatives, such as 2-heptyl-4-hydroxyquinoline-N-oxide (HQNO), aurachin C, and floxacrine, have been reported as effective cytochrome *bc*_1_ complex inhibitors, the bioactivity of these products is not ideal, presumably due to their low bioavailability in tissues, particularly their poor solubility and low mitochondrial accumulation. In order to overcome the drawbacks of these compounds and develop their use as agricultural fungicides acting by cytochrome *bc*_1_ inhibition, in this study, three novel mitochondria-targeting quinolone analogs (mitoQNOs) were designed and synthesized by conjugating triphenylphosphonium (TPP) with quinolone. They exhibited greatly enhanced fungicidal activity compared to the parent molecule, especially mitoQNO_11_, which showed high antifungal activity against *Phytophthora capsici* and *Sclerotinia sclerotiorum* with EC_50_ values of 7.42 and 4.43 μmol/L, respectively. In addition, mitoQNO_11_ could inhibit the activity of the cytochrome *bc*_1_ complex of *P. capsici* in a dose-dependent manner and effectively depress its respiration and ATP production. The greatly decreased mitochondrial membrane potential and massively generated reactive oxygen species (ROS) strongly suggested that the inhibition of complex III led to the leakage of free electrons, which resulted in the damage of the pathogen cell structure. The results of this study indicated that TPP-conjugated QNOs might be used as agricultural fungicides by conjugating them with TPP.

## 1. Introduction

**Figure 1:**
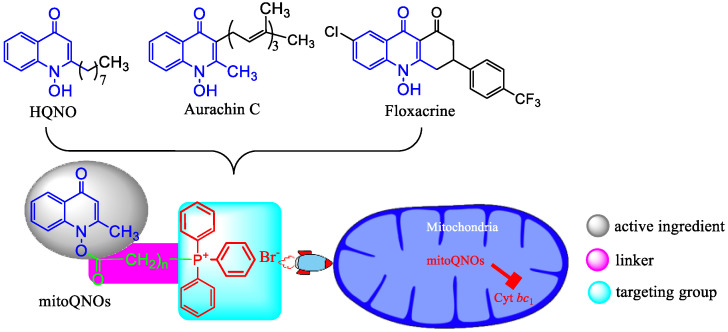
Design strategy of mitoQNOs.

Since the first quinolone derivative nalidixic acid was reported in 1962, more than 100,000 quinolone derivatives have been synthesized, which has been one of the hot spots in drug development [1]. Quinolones have demonstrated antiviral, antituberculotic, anticancer, fungicidal, and other beneficial biological activities and act on different targets [2,3]. Among them, 2-heptyl-4-hydroxyquinoline-N-oxide (HQNO, Figure 1) is reported to be a cytochrome *bc*_1_ complex (complex III) inhibitor, mainly used against *Staphylococcus aureus*. As an analog in the ubiquinone, it can occupy the quinone reduction site (Q_i_ site) of cyt. *bc*_1_ complex to block electron transfer of electron transfer chain (ETC) [3,4]. Aurachin C is a natural product isolated from *Stigmatella aurantiaca* strain Sg a15 [5] and its analogs are usually effective inhibitors of Q_i_ site [6,7], whereas floxacrine is an antimalarial drug also acting as cyt. *bc*_1_ complex inhibitor [8,9]. Because all of these compounds contain a 1-hydroxy-4-quinolone (QNO) framework, which is structurally similar to ubiquinone in mitochondria, they exhibit good enzyme inhibitory activity on cyt. *bc*_1_ complex. However, most of them show poor activity in vivo, presumably due to their poor solubility and mitochondrial accumulation [10,11]

The cyt. *bc*_1_ complex, as a central component of the mitochondrial respiratory chain, mainly catalyzes the transfer of electrons from hydroquinone to the water-soluble electron receptor cytochrome c, accompanied by transmembrane transferring of protons [12]. In agricultural production, fungicides targeting complex III have been the most successful crop protection products for the past 30 years [6,13]. However, frequent and extensive field applications have caused serious resistance of phytopathogens to these fungicides, and new fungicides or resistance management strategies are urgently needed. Moreover, so far there are no reports on the effects and applications of 1-hydroxy-4-quinolone derivatives against plant pathogenic fungi.

Cations such as triphenylphosphonium (TPP) can be accumulated rapidly and efficiently in mitochondria due to their excellent affinity to the negatively charged mitochondrial membrane and high lipophilicity; therefore, TPP is frequently used in mitochondria-targeting drug research [14]. By conjugating TPP to a traditional amide fungicide, we obtained novel mitochondria-targeting succinate dehydrogenase (SDH) inhibitors with high efficiency and low resistance risk, which proved their potential in the development of new ETC inhibitors [15]. To overcome the drawbacks of poor solubility and less efficiency in mitochondrial accumulation of MQNO, the purposes of this study are to (i) synthesize mitochondria-targeted quinolones, termed mitoQNOs, which consist of an active ingredient, a linker, and a mitochondria-targeting group TPP (Figure 1); (ii) investigate the antifungal efficacy of these compounds; and (iii) derive mechanistic insights into the action of mitoQNOs.

## 2. Materials and Methods

**Instrumentation and Materials.** The chemicals and reagents used were obtained from commercial sources without further purification. The ^1^H NMR (300 MHz) and ^13^C NMR (75 MHz) spectra were obtained on a Bruker AVANCE DPX300 spectrometer (Bruker Corp., Billerica, MA, USA) using tetramethylsilane as the internal standard. High-resolution mass spectra (HRMS) were obtained on an Agilent-ESI-Q-TOF spectrometer (Agilent Tech. Inc., Lexington, CA, USA). FL × 800 Fluorescent microplate reader (BioTek, Winooski, VT, USA), PowerWave XS2 microplate reader (BioTek, Winooski, VT, USA), and Olympus CCD−DP72 fluorescent microscope (Olympus Co., TKY, Japan) were used for fluorescence measurement. Melting point was recorded on a Yanagimoto MFG apparatus, and the thermometer was uncorrected.

**Synthetic Procedures** **.**

Synthesis of compound **1**. A mixture of 75 mmol of aniline, 3.5 mL of acetic acid, and 75 mmol of ethyl acetoacetate in 90 mL of toluene was refluxed to dehydration via azeotropic distillation. After 6 h, the reaction was completed, and the mixture was concentrated to dryness in vacuo to afford **1** as crude product, which was directly used in the next step without further purification. 

Synthesis of compound **2**. Diphenyl ether (70 mL) was added to crude **1**, and the mixture was allowed to reflux for 10 min. After cooling, 200 mL ethyl acetate was added, and the precipitated solid was collected and washed with ethyl acetate. An amount of 8.7 g of compound **2** was obtained as white powder with 73% yield, mp. 233.8–234.4 °C. ^1^H NMR (300 MHz, DMSO-*d*_6_) δ 10.65 (s, 1H), 8.37–8.23 (m, 1H), 8.11 (qd, *J* = 6.8, 3.3 Hz, 1H), 7.85–7.75 (m, 1H), 7.23 (s, 1H), 2.82 (s, 2H).

Synthesis of compound **3**. Potassium tert-butoxide (100 mmol) was added to a solution of 50 mmol compound **2** in 100 mL THF and the mixture was stirred for 1 h at room temperature. The reaction mixture was cooled in an ice bath, 52 mmol of dimethylcarbamic chloride was dropped in, and the reaction was continued for 2 h below 10 °C. It was extracted with ethyl acetate (3 × 100 mL) and the combined organic phase was dried over anhydrous sodium sulfate and then filtered. The filtrate was evaporated to dryness under reduced pressure, and the residue was purified by silica gel chromatography to afford **3** as white powder with 93% yield, mp. 129.6–131.8 °C. ^1^H NMR (300 MHz, DMSO-*d*_6_) δ 10.65 (s, 1H), 8.37–8.23 (m, 1H), 8.11 (qd, *J* = 6.8, 3.3 Hz, 1H), 7.85–7.75 (m, 1H), 7.23 (s, 1H), 2.82 (s, 2H).

Synthesis of compound **4**. Compound **3** (40 mmol) was dissolved in 150 mL of dichloromethane, then 60 mmol of 85% meta-chloroperbenzoic acid (MCPBA) was added slowly under ice-bath cooling, and the reaction was continued for 5 h at room temperature. It was washed with saturated aqueous sodium bicarbonate (2 × 60 mL), and the combined organic phase was dried over anhydrous sodium sulfate and then filtered. The filtrate was evaporated to dryness under reduced pressure, and the residue was purified by silica gel chromatography to afford **4** as brown powder with 90% yield, mp. 236.2–237.8 °C. ^1^H NMR (300 MHz, CDCl_3_) δ 7.99 (dd, *J* = 25.2, 7.9 Hz, 1H), 7.74–7.64 (m, 1H), 7.48 (t, *J* = 7.2 Hz, 1H), 7.27 (s, 1H), 3.23 (s, 3H), 3.07 (s, 3H), 2.74 (s, 3H).

Synthesis of compound **5**. Compound **4** (30 mmol) was hydrolyzed with 30 mL of 5 mol/L potassium hydroxide at room temperature for 2 h. It was adjusted pH = 2 with 5 mol/L hydrochloride and the precipitated grey solid was collected and dried, obtaining 5 g compound **5** with 96% yield, mp. 266.0–267.5 °C. ^1^H NMR (300 MHz, DMSO-*d*_6_) δ 9.76 (s, 1H), 8.37–8.22 (m, 2H), 8.16–8.03 (m, 1H), 7.80 (t, *J* = 7.3 Hz, 1H), 7.22 (s, 1H), 2.81 (s, 3H).

General synthetic procedure of compound **6**. A mixture of 8.6 mmol of compound **5**, 25.8 mmol trimethylamine, and 50 mL dichloromethane was stirred for 30 min in an ice bath. ω-bromoaliphatic acyl chloride (9.04 mmol) was added slowly and the reaction was continued for 2 h at ambient temperature. The mixture was extracted with dichloromethane (3 × 100 mL), and the combined organic phase was dried over anhydrous sodium sulfate and then filtered. The filtrate was evaporated to dryness under reduced pressure, and the residue was purified by silica gel chromatography to afford **6**.

Data for **6**–**1**. White powder, mp. 136.0–138.5 °C, yield 89%. ^1^H NMR (300 MHz, CDCl_3_) δ 8.30 (dd, *J* = 8.1, 1.3 Hz, 1H), 7.57 (ddd, *J* = 8.5, 7.3, 1.4 Hz, 1H), 7.30 (t, *J* = 7.6 Hz, 1H), 7.12 (d, *J* = 8.5 Hz, 1H), 3.39 (t, *J* = 6.5 Hz, 2H), 2.73 (td, *J* = 7.3, 2.5 Hz, 2H), 2.22 (s, 3H), 1.96–1.74 (m, 4H), 1.64–1.50 (m, 2H). ^13^C NMR (75 MHz, CDCl_3_) δ 176.73, 169.98, 148.14, 138.64, 132.32, 126.17, 124.77, 123.66, 112.08, 108.86, 32.91, 31.69, 30.55, 27.17, 23.32, 17.46. HRMS (ESI) for (C_16_H_18_BrNO_3_) ([M + H]^+^) 352.0544.

Data for **6**–**2**. White powder, mp. 144.3–146.0 °C, yield 86%. ^1^H NMR (300 MHz, CDCl_3_) δ 8.27 (dd, *J* = 8.1, 1.3 Hz, 1H), 7.54 (ddd, *J* = 8.5, 7.2, 1.5 Hz, 1H), 7.31–7.20 (m, 1H), 7.10 (d, *J* = 8.4 Hz, 1H), 6.02 (s, 1H), 3.33 (t, *J* = 6.7 Hz, 2H), 2.67 (td, *J* = 7.4, 2.3 Hz, 2H), 2.19 (s, 3H), 1.84–1.66 (m, 4H), 1.49–1.24 (m, 6H). ^13^C NMR (75 MHz, CDCl_3_) δ 176.65, 170.15, 148.20, 138.62, 132.27, 126.05, 124.71, 123.58, 112.13, 108.75, 33.54, 32.14, 30.60, 28.42, 27.80, 27.40, 23.99, 17.41. HRMS (ESI) for (C_18_H_22_BrNO_3_) ([M + H]^+^) 380.0859.

Data for **6**–**3**. White powder, mp. 163.0–164.5 °C, yield 83%. ^1^H NMR (300 MHz, DMSO-*d*_6_) δ 8.33 (dd, *J* = 8.1, 1.4 Hz, 1H), 7.65–7.53 (m, 1H), 7.36–7.29 (m, 1H), 7.16 (d, *J* = 8.5 Hz, 1H), 3.38 (t, *J* = 6.8 Hz, 2H), 2.72 (td, *J* = 7.4, 1.8 Hz, 2H), 2.25 (s, 3H), 1.81 (dq, *J* = 13.7, 7.0 Hz, 4H), 1.46–1.22 (m, 12H). ^13^C NMR (75 MHz, CDCl_3_) δ 176.74, 170.18, 148.11, 138.69, 132.20, 126.20, 124.81, 123.58, 112.07, 108.86, 33.67, 32.39, 30.75, 28.91, 28.83, 28.67, 28.29, 27.71, 24.19, 17.42. HRMS (ESI) for (C_21_H_29_BrNO_3_) ([M + H]^+^) 422.1321.

General synthetic procedure of mitoQNOs. To a mixture of 3 mmol of compound **6**, 3.6 mmol of triphenylphosphine and 0.14 mmol potassium iodide 50 mL acetonitrile were added at argon atmosphere and the mixture was allowed to reflux for 12 h. The solvent was evaporated in vacuo and the residue was purified by silica gel chromatography to afford mitoQNOs.

Data for mitoQNO_6_. Viscose yellow oil, yield 71%. ^1^H NMR (300 MHz, CDCl_3_) δ 8.23 (d, *J* = 8.2 Hz, 1H), 7.85–7.59 (m, 16H), 7.30 (s, 1H), 7.22 (t, *J* = 7.7 Hz, 1H), 6.05 (s, 1H), 3.78–3.52 (m, 2H), 2.99–2.75 (m, 2H), 2.19 (s, 3H), 1.96–1.56 (m, 6H). ^13^C NMR (75 MHz, CDCl_3_) δ 176.77, 170.19, 148.88, 138.51, 134.84, 134.81, 133.37, 133.24, 133.15, 133.10, 132.91, 130.33, 130.17, 125.83, 124.46, 123.78, 118.14, 117.00, 112.82, 108.63, 30.62, 29.40, 29.19, 23.41, 22.90, 21.97, 21.92, 17.90. ^31^P NMR (121 MHz, CDCl3) δ 25.02. HRMS (*m*/*z*) calcd. for C_34_H_33_NO_3_P^+^ [M]^+^ 534.2193, found 534.2191.

Data for mitoQNO_8_. Yellow solid, m.p. 63.6–64.2 °C, yield 84%. ^1^H NMR (300 MHz, CDCl_3_) δ 8.30 (d, *J* = 7.2 Hz, 1H), 7.89–7.61 (m, 16H), 7.34 (s, 1H), 7.31–7.20 (m, 1H), 6.12 (s, 1H), 3.85–3.65 (m, 2H), 2.97–2.68 (m, 2H), 2.22 (s, 3H), 1.92–1.38 (m, 10H). ^13^C NMR (75 MHz, CDCl_3_) δ 176.76, 170.32, 148.75, 138.57, 134.71, 134.67, 133.31, 133.18, 133.08, 132.69, 130.23, 130.06, 125.95, 124.53, 123.72, 118.42, 117.28, 112.55, 108.77, 30.68, 29.68, 29.47, 29.26, 28.12, 27.94, 23.81, 22.68, 22.15, 22.09, 22.01, 17.58. ^31^P NMR (121 MHz, CDCl3) δ 24.93. HRMS (*m*/*z*) calcd. for C_36_H_37_NO_3_P^+^ [M]^+^ 562.2506, found 562.2500.

Data for mitoQNO_11_. Yellow solid, m.p. 85.3–86.2 °C, yield 82%. ^1^H NMR (300 MHz, CDCl_3_) δ 8.08 (d, *J* = 8.0 Hz, 1H), 7.69–7.52 (m, 15H), 7.51–7.45 (m, 1H), 7.15 (t, *J* = 7.6 Hz, 1H), 7.09 (d, *J* = 8.5 Hz, 1H), 5.87 (s, 1H), 3.44–3.30 (m, 2H), 2.64 (td, *J* = 7.3, 2.3 Hz, 2H), 2.13 (s, 3H), 1.71–1.58 (m, 2H), 1.47 (s, 4H), 1.34–1.20 (m, 2H), 1.20–1.01 (m, 8H). ^13^C NMR (75 MHz, CDCl_3_) δ 176.57, 170.35, 148.30, 138.57, 134.78, 134.74, 133.18, 133.05, 132.40, 130.25, 130.08, 125.75, 124.59, 123.51, 118.11, 116.97, 112.35, 108.55, 30.80, 30.08, 29.87, 28.60, 28.56, 28.45, 24.03, 22.89, 22.22, 22.15, 22.09, 17.59. ^31^P NMR (121 MHz, CDCl3) δ 25.05. HRMS (*m*/*z*) calcd. for C_39_H_43_NO_3_P^+^ [M]^+^ 604.2975, found 604.2971.

**In vitro fungicidal activity assay.** The in vitro fungicidal activity of the mitoQNOs was determined by mycelium growth rate method, and boscalid (complex II inhibitor) and kresoxim-methyl (cyt. *bc*_1_ complex inhibitor) were used as positive controls. When the mycelium in the control group was fully grown in the whole Petri dish, the diameter of the colony in each treating Petri dish was recorded with the criss-cross method, and the inhibition rate of mycelial growth was calculated as follows:

Mycelial growth diameter (mm) = mean diameter (measured three times)–5.0 (diameter of fungus plug).

Mycelial growth inhibition rate (%) = [(mycelium growth diameter of the control group–mycelium growth diameter of the treatment group)/mycelium growth diameter of the control group] × 100%.

The median effect concentration (EC_50_) values were calculated according to the linear regression of colony diameter on log-transformed fungicide concentrations. The experiment was repeated three times in each group.

**Determination of mycelium respiration.** Active mycelial cakes were obtained by punching holes at the edge of the activated fungi, and 6–10 pieces of fungus plugs were added to 100 mL of PDB medium. Then the PDB medium was cultured in constant temperature shaker at 25 °C and 160 rpm for 48 h. The hyphae were filtered by vacuum pump, and hyphal respiration was measured by Clark oxygen electrode [16]. The experiment was repeated three times in each group.

**Mitochondrial complex III inhibition assay.** Hyphal mitochondria were extracted by differential centrifugation. After three times freeze-thaw, samples were treated with different concentrations of mitoQNO_11_ and pyrimorph, then complex III activity was detected by complex III activity assay kit (*Genmed Scientifics Inc.*, Plymouth, MN, USA, GMS50009) [17]. The experiment was repeated three times in each group.

**Measurement of ATP level in mycelium of *P. capsici*.** Hyphae were cultured for 48 h in PDB medium and continued to grow for 3 h after adding fungicides. Then, the hyphae were filtered out in vacuo and cut into 10 mg-sized hyphae with a knife. A total of 0.3 mL of PBS (10 mM) and 0.3 mL pyrolysis solution were added. Ultrasound breaker (G800R3) was used to break the structure of hyphae. The supernatant was centrifuged at 4 °C, 12,000 rpm for 10 min, then taking the supernatant. The ATP level in the supernatant was assessed using the adenosine 5′-triphosphate (ATP) bioluminescent assay kit (*Beyotime*, S0027) and a fluorescence microplate reader (FL × 800) [18]. The experiment was repeated three times in each group.

**Determination of mitochondrial membrane potential (MMP).** *JC-1* probe was employed to evaluate the MMP in *P. capsici* strain [19]. Briefly, after 48 h of culturing in PDB, hyphae continued to grow for 3 h after adding fungicides. Then the hyphae were incubated with an equal volume of serum-free medium containing *JC-1 dye* (5 mg/L) at 25 °C for 30 min and rinsed twice with 1% glucose solution. Finally, images were taken in the green and red fluorescence channel by fluorescence microscope imaging (*Olympus CCD*−*DP72* and *Leica SP5*), and the data analysis of fluorescence intensity was performed by Image J. The experiment was repeated three times in each group.

**Measurement of intracellular ROS level.** Intracellular ROS level was evaluated in conidia of *P. capsici* using the fluorescent probe 2′,7′-dichlorofluorescin diacetate (DCFH-DA) [19]. DCFH-DA is a non-fluorescent, lipophilic, and non-ionic compound capable of diffusing and crossing the cell membrane into the cytoplasm. Then DCFH−DA is deacetylated by intracellular esterases, producing 2′,7′-dichlorofluorescin (DCFH), a non-fluorescent and membrane-impermeable compound that reacts with intracellular ROS to generate a green fluorescent 2′,7′-dichlorofluorescin (DCF). Briefly, hyphae after 48 h of culture in PDB medium continued to grow for 0.5 h after adding 1 μL DCFH-DA (10 mmol). After rinsing twice with 1% glucose solution, the tested compounds were added and incubation was continued for 3 h. Finally, images were taken in the green fluorescence channel by fluorescence microscope imaging (*Olympus CCD*−*DP72*), and the data analysis of fluorescence intensity was performed by Image J. The experiment was repeated three times in each group.

## 3. Results

### Subsection

**Scheme 1:**
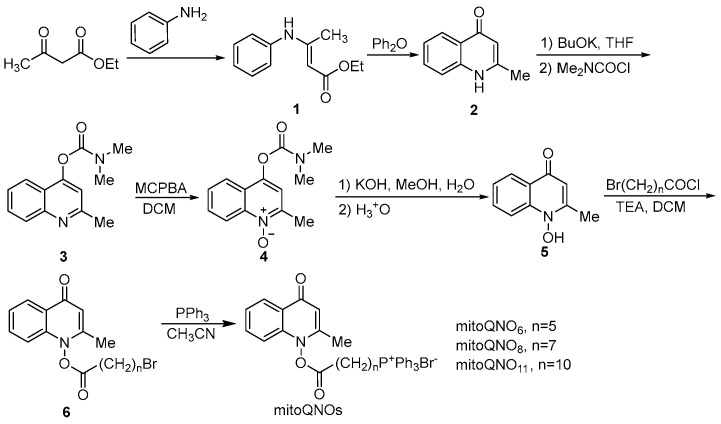
The synthetic pathway of mitoQNOs.

- **Chemistry.** The key intermediate 2-methyl-1-hydroxy-4(1H)-quinolone (**5**) was prepared with a similar method described by Cross and Cornforth [20,21]. It was then acylated to give **6**, which reacted with triphenylphosphine by a nucleophilic substitution to afford mitoQNOs (Scheme 1)

- **MitoQNO_11_ exhibited significantly enhanced fungicidal activity.** Results concerning fungicidal activities of mitoQNOs against 11 plant pathogenic fungi, preliminarily determined at a concentration of 70 μmol, are shown in Table 1. As we previously described for SDH inhibition [15], the activity of mitoQNOs was also enhanced with the increase in chain length of the TPP-conjugated linker. For example, the inhibition rates of mitoQNO_6_, mitoQNO_8_, and mitoQNO_11_ against *S. sclerotiorum* were 19.86%, 45.44%, and 91.47%, respectively. This is mainly because of the increase in liposolubility, which makes it easier to pass through the membrane (Table 1). Meanwhile, it was also found that mitoQNO_11_ exhibited excellent fungicidal activity against *P. aphanidermatum*, *P. capsica*, and *B. cinerea*, and the inhibition rates were 75.63%, 87.50%, and 78.88%, respectively. Compared with the lead molecule MQNO, mitoQNO_11_ showed significantly enhanced fungicidal activity against all tested pathogenic fungi.

**Table 1:** In vitro fungicidal activity of title compounds.

Pathogen ^1^	Inhibition Rate (70 μmol/L, %)					
	MQNO	MitoQNO_6_	MitoQNO_8_	MitoQNO_11_	Boscalid	Kresoxim-Methyl
PA	15.21 ± 1.06	0 ± 0.59	0 ± 0.51	75.63 ± 0.88	9.79 ± 1.06	81.25 ± 7.14
PC	10.90 ± 0.54	2.70 ± 0.31	29.96 ± 1.66	87.5 ± 1.32	17.11 ± 1.25	30.19 ± 0.54
SS	14.75 ± 1.59	19.86 ± 2.63	45.44 ± 2.65	91.47 ± 2.68	95.31 ± 0.60	40.32 ± 0.60
BC	13.01 ± 7.97	0 ± 0.59	0 ± 0.59	78.88 ± 5.33	92.05 ± 0.59	30.78 ± 0.30
BB	18.10 ± 3.85	0 ± 0.54	0 ± 0.31	41.67 ± 0.59	54.79 ± 1.93	72.08 ± 0.78
ET	2.15 ± 0.33	2.86 ± 0.88	4.73 ± 0.33	24.86 ± 0.57	95.32 ± 0.66	26.5 ± 0.66
CA	2.95 ± 0.55	0 ± 0.63	3.61 ± 1.09	19.23 ± 0.63	94.65 ± 1.09	50.47 ± 1.09
FO	5.18 ± 0.83	5.18 ± 1.14	6.29 ± 0.87	49.58 ± 1.26	38.20 ± 0.32	59.39 ± 0.63
RS	5.42 ± 1.18	0.42 ± 0.59	0.42 ± 0.59	45.00 ± 1.02	87.08 ± 0.59	72.92 ± 0.29
PI	0 ± 0.65	5.55 ± 1.72	7.15 ± 0.15	15.64 ± 3.24	57.36 ± 1.49	63.09 ± 0.32
CL	9.35 ± 2.03	3.12 ± 1.48	6.00 ± 0.68	15.83 ± 1.02	40.53 ± 1.48	1.44 ± 1.02
logP ^2^	0.49	5.65	6.66	8.16	4.64	4.13

^1.^ PA = *Pythium aphanidermatum*; PC = *Phytophthora capsici*; SS = *Sclerotinia sclerotiorum*; BC = *Botrytis cinerea*; BB = *Botryosphaeria berengeriana*; ET = *Exserohilum turcicum*; CA = *Cercospora arachidicola Hori*; FO = *Fusarium oxysporum f.sp. vasinfectum*; RS = *Rhizoctonia solani*; PI = *Phytophthora infestans*; CL = *Colletotrichum lagenarium*. ^2.^ Calculated by Molinspiration Cheminformatics 2019.

- In order to verify the effectiveness of mitoQNO_11_, we further determined its precision toxicity against four pathogenic fungi (Table 2). Compared with the selected fungicides that act on the mitochondrial respiratory chain, mitoQNO_11_ showed good inhibitory effects towards the four tested strains. The EC_50_ values for *P. capsici*, *P. aphanidermatum*, *B. cinereal*, and *S. sclerotiorum* were 7.42, 51.42, 21.73, and 4.43 μmol/L, respectively. Especially, mitoQNO_11_ exhibited excellent fungicidal activity against *P. capsici*, a pathogenic fungus causing phytophthora blight of pepper, which has become increasingly serious in recent years [22].

**Table 2:** Precision toxicity of mitoQNO_11_ against four pathogenic fungi.

Compound	EC_50_ (μmol/L)			
	*P. capsici*	*S. sclerotiorum*	*B. cinerea*	*P. aphanidermatum*
MQNO	>140	>140	>140	>140
mitoQNO_11_	7.42 ± 0.73	4.43 ± 1.51	21.73 ± 1.71	51.42 ± 3.67
boscalid	>140	0.83 ± 0.07	6.79 ± 0.64	>140
kresoxim-methyl	>140	71.41 ± 18.88	>140	51.56 ± 18.63
pyrimorph	3.77 ± 0.19	74.33 ± 8.59	>140	>140

**Figure 2:**
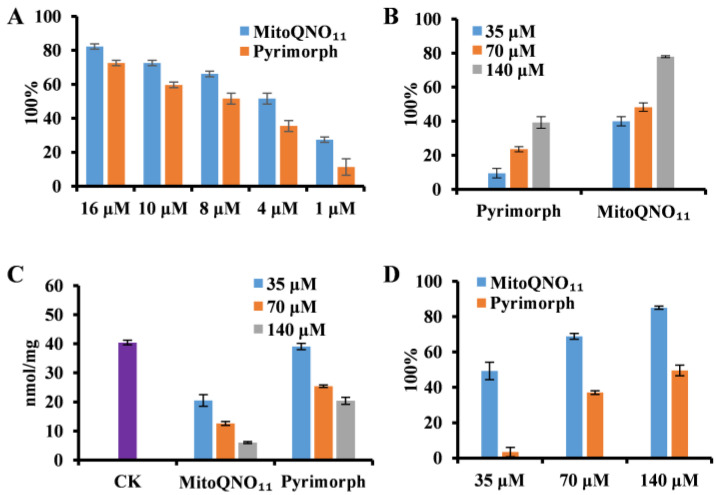
Influences comparison of mitoQNO_11_ with pyrimorph on the respiration and ATP production of *P. capsici*. (**A**) Column chart depicting the inhibition rate of cytochrome *bc*_1_ complex of *P. capsica*. (**B**) Column chart depicting the respiratory inhibiting rate of *P. capsica*. (**C**) Changes of ATP content in hyphae after treatment with mitoQNO_11_. (**D**) ATP inhibitory rate of mitoQNO_11_ and pyrimorph. Data points correspond to the mean of three independent experiments ± standard error of the mean.

**Figure 3:**
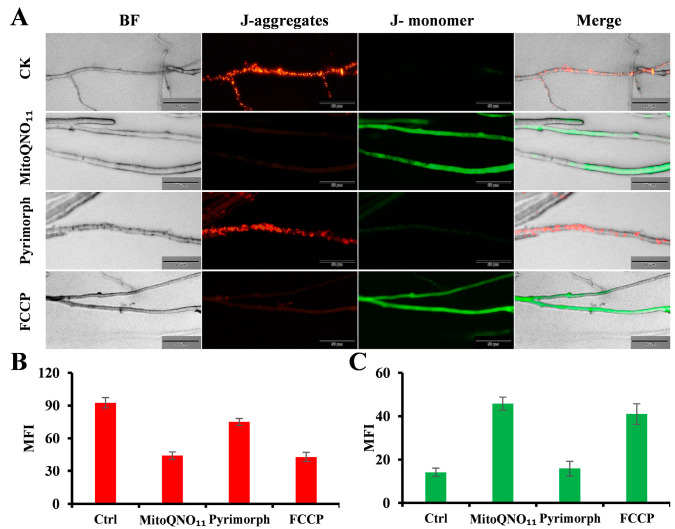
Effect of mitoQNO_11_ on mitochondrial membrane potential as monitored by *JC-1* staining. *JC-1* aggregates accumulate in the mitochondrial matrix with high ∆Ψm, emitting orange-red fluorescence; upon depolarization of the mitochondrial membrane, *JC-1* aggregates dissipate into the cytoplasm as monomers emitting green fluorescence. (**A**) *JC-1* staining was observed by fluorescence microscopy after treatment with mitoQNO_11_, pyrimorph, and FCCP at 20 μmol/L. Scale bar 50 μm. (**B**) Change in mean fluorescence intensity (MFI) of red fluorescence (JC-1 aggregates) after treatment with mitoQNO_11_, pyrimorph, and FCCP, respectively, at 20 μmol/L. (**C**) Change in mean fluorescence intensity of green fluorescence (*JC-1* monomer) after treatment with mitoQNO_11_, pyrimorph, and FCCP at 20 μmol/L. Data points correspond to the mean of three independent experiments ± standard error of the mean.

**Figure 4:**
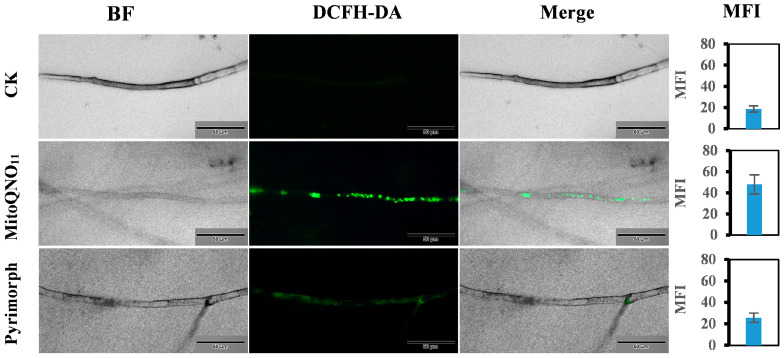
Effect of MitoQNO_11_ on ROS was monitored by DCFH-DA staining and observed by fluorescence microscopy after treatment with 20 μmol/L MitoQNO_11_ or 20 μmol/L pyrimorph for 3 h at 25 °C. Scale bar, 50 μm. MFI: mean fluorescence intensity. Data points correspond to the mean of three independent experiments ± standard error of the mean.

**Figure 5:**
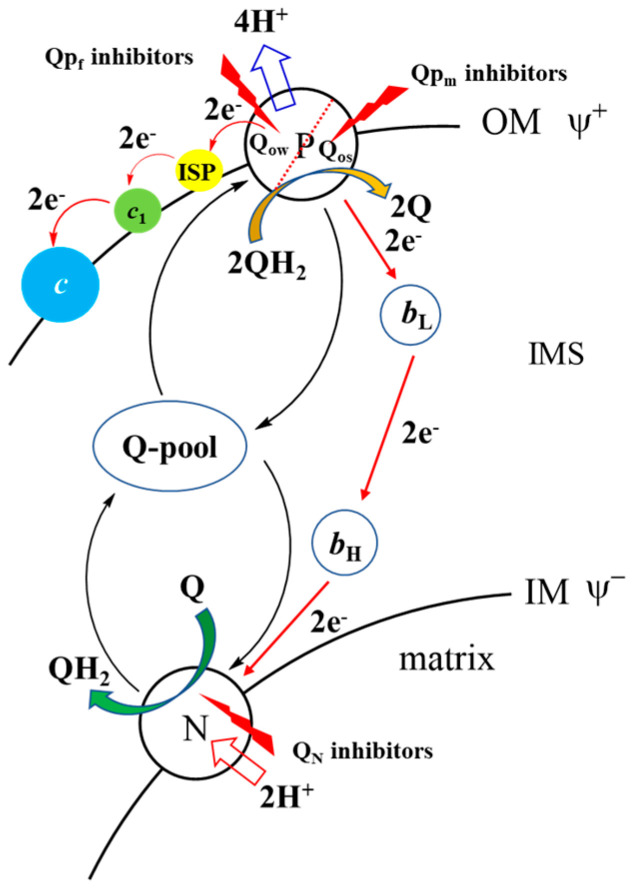
Schematic diagram of working principle of Complex III and targeting site of inhibitors.

- **MitoQNO_11_ effectively inhibited the activity of cytochrome *bc*_1_ complex.** Most of the 1-hydroxy-4-quinolone derivatives were reported as cytochrome *bc*_1_ complex inhibitors, so the effect of mitoQNO_11_ on *bc*_1_ complex was also investigated. As shown in Figure 2A, mitoQNO_11_ exhibited more effective inhibitory ability against cytochrome *bc*_1_ complex compared to pyrimorph (a potent fungicide against *P. capsici* mainly acting on cell wall syntheses well as on *bc*_1_ complex) [23]. The IC_50_ values of mitoQNO_11_ and pyrimorph were 3.33 and 6.89 μmol/L, respectively (Table S1). Therefore, mitoQNO_11_ is a highly potent inhibitor of *bc*_1_ complex, even though it might have other action mechanisms.

- **MitoQNO_11_ strongly suppressed mycelium respiration.** As an important part of ETC, the inhibition of the *bc*_1_ complex results in the inhibition of mitochondria respiration. Therefore, the influence of mitoQNO_11_ on the mycelium respiration of *P. capsici* was measured. As shown in Figure 2B, at a concentration of 35 μmol/L, the inhibition rate of mitoQNO_11_ was 4.2 times higher than that of pyrimorph, and the inhibition was dose-dependent.
- **MitoQNO_11_ strongly inhibited ATP generation.** Inhibition of respiration implies a significant reduction in ATP synthesis by the pathogen. Therefore, we monitored the changes in cellular ATP of *P. capsici* after drug treatment by firefly luciferase. After three hours of treatment at a concentration of 35 μmol/L, the ATP level in the mitoQNO_11_ group was reduced by 50%, whereas in the pyrimorph group, it was reduced by less than 10% (Figure 2C); the inhibition was found to be dose-dependent. Even at 140 μmol/L, the ATP level in the pyrimorph group decreased by less than half, whereas the inhibition rate of mitoQNO_11_ reached to 85.08% after three hours of treatment at the same concentration (Figure 2D).
- **MitoQNO_11_ strongly reduced mitochondrial membrane potential.** The integrity of the ETC function is the critical element in maintaining MMP (∆Ψm). Next, we investigated the impact of mitoQNO_11_ on ∆Ψm by using a cationic dye *JC-1*, a potent indicator of MMP changes due to the formation of red fluorescent ‘J-aggregates’.
- The mycelium of *P. capsici* was treated with mitoQNO_11_, pyrimorph, or FCCP (a well-known uncoupler that can easily cause a reduction in mitochondrial membrane potential), respectively, at 20 μmol/L for 3 h at 25 °C, followed by staining with *JC-1*. As shown in Figure 3A, in the control group the granular mitochondria in hyphae emitted red fluorescence, whereas no obvious green fluorescence was observed, indicating high ∆Ψm. In the mitoQNO_11_ group, we observed the obvious disappearance of red fluorescence and an enhancement of green fluorescence, indicating the decrease in ∆Ψm. By analyzing its mean fluorescence intensity (Figure 3B), it was found that the intensity of red fluorescence decreased by half compared to the control group, whereas the intensity of green fluorescence was three times higher than that of the control group (Figure 3C). However, there was no decrease in red fluorescence in pyrimorph treatment group compared with the control group, and similar results were obtained by analyzing the mean fluorescence intensity. As a positive control, after treatment with FCCP, the obvious disappearance of red fluorescence and the enhancement of green fluorescence were also observed (Figure 3A,B). These results indicated that the MMP of *P. capsici* was strongly reduced by mitoQNO_11_.

- **MitoQNO_11_ strongly induced ROS production.** Mitochondrial dysfunction is closely related to the excessive reactive oxygen species (ROS) and the respiratory chain is the main source of ROS in eukaryote. The inhibition of the respiratory chain can lead to the disruption of oxidative stress balance. Then we examined the changes of intracellular ROS level using an oxidation-sensitive probe, 2′,7′-dichlorofluorescin diacetate (DCFH-DA).
- As shown in the control group, no fluorescence signal was observed (Figure 4). However, obvious green fluorescence was observed in the mitoQNO_11_ group, and we could see bright green fluorescence distributed in dots on the hyphae. In stark contrast, there was no obvious green fluorescence in the pyrimorph group. By analyzing mean fluorescence intensity, it was found that the green fluorescence intensity doubled after mitoQNO_11_ treatment. These observations suggested that mitoQNO_11_ has a strong capability to induce ROS generation in phytopathogens. Considering that the cytochrome *bc*_1_ complex is one of the main sources of mitochondrial ROS and that 1-hydroxy-4-quinolone derivatives mainly target the Q_i_ site [24,25,26], we propose that the primary mechanism of action of mitoQNO_11_ is that it accumulates in mitochondria and binds to the Q_i_ site of cytochrome *bc*_1_ complex, thus leading to the leakage of electrons differentiated from the Q_o_ site, resulting in the production of large amounts of ROS (Figure 5).

## 4. Discussion

In summary, in order to remedy the defects of 1-hydroxy-4-quinolones, three derivatives of QNO termed mitoQNOs were designed and synthesized by conjugation with triphenylphosphonium. The results suggested that the fungicidal activity of mitoQNOs increased with the increase in alkyl chain length, which is consistent with the findings in TPP-driven SDHIs [15]. MitoQNO_11_ exhibited excellent fungicidal activity against *P. capsici*, *P. aphanidermatum*, *B. cinereal*, and *S. sclerotiorum*, which has been confirmed by further precise toxicity experiments. Extensive studies suggested that mitoQNO_11_ is a strong cytochrome *bc*_1_ complex inhibitor, leading to a series of subsequent effects, such as inhibition of hyphal respiration and the generation of ATP, and reduction in membrane potential. Moreover, the vast generation of ROS after treatment with mitoQNO_11_ was also observed. This is believed to depend on the inhibition of the Q_i_ site which resulted in the blocking of electron transfer from the reduced cytochrome *b_5_*_62_ to the oxidized quinone, thus leading to the accumulation of semiquinone radicals at Q_o_ sites and causing electron leakage to generate large amounts of ROS [26,27]. Furthermore, when the cytochrome *bc*_1_ complex was inhibited, the blockage of the respiratory chain could cause the inhibition of hyphal respiration; meanwhile, the blockage of electron transfer could lead to the reduction in mitochondrial membrane potential. On the other hand, due to the coupling of the respiratory chain and oxidative phosphorylation, ATP synthesis was suppressed. All of these results confirm that targeting bioactive molecules to mitochondria by conjugating the lipophilic TPP cation represents an effective fungicidal strategy. However, despite the reasonable analysis of the experimental results, the actual mechanism of action may be much more complicated. For example, the Q_i_ site is probably not the only site of MitoQNO_11_, and other active sites which use Q/QH_2_ as the substrate are also potential targets. In addition, the damage to the cell membrane caused by the surface effect of TPP itself should also be a concern. Therefore, to accurately describe its action mechanism, more experimental evidence is needed.

## Data Availability

Not applicable.

## Supplementary Materials

The following supporting information can be downloaded at: https://www.mdpi.com/article/10.3390/jof9060685/s1, Figure S1: Spectra of title compounds; Table S1: IC_50_ value of MitoQNO_11_ and pyrimorph on cytochrome *bc*_1_ complex of *P. capsici*.
