# Supplementary material for: Triphenylphosphonium (TPP)-Conjugated Quinolone Analogs Displayed Significantly Enhanced Fungicidal Activity Superior to Its Parent Molecule
Source: J Fungi (Basel). 2023 Jun 19;9(6):685. doi: 10.3390/jof9060685 (PMC10305039; doi:10.3390/jof9060685)
Supplement: Supplementary file 1 [file jof-09-00685-s001.zip › jof-2443095-supplementary material.pdf]

**Supplementary Table S1.** $IC_{50}$ value of $MitoQNO_{11}$ and pyrimorph on cytochrome $bc_1$ complex of *P. capsici*.

| | $EC_{50}$ | $R^2$ | Toxicity regression equations |
|---|---|---|---|
| $MitoQNO_{11}$ | 3.33±0.29 | 0.989 | Y=1.24X-0.65 |
| Primorph | 6.89±2.16 | 0.996 | Y=1.48X-1.24 |

**Supplementary Figure S1.** Spectra of title compounds.

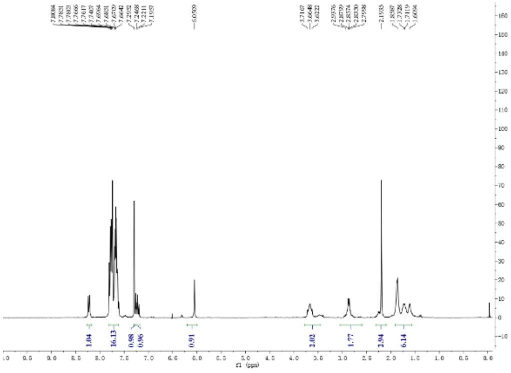

$^1H$ NMR of $mitoQNO_6$

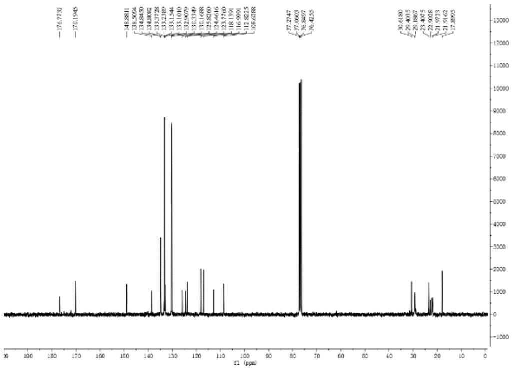

$^{13}C$ NMR of $mitoQNO_6$

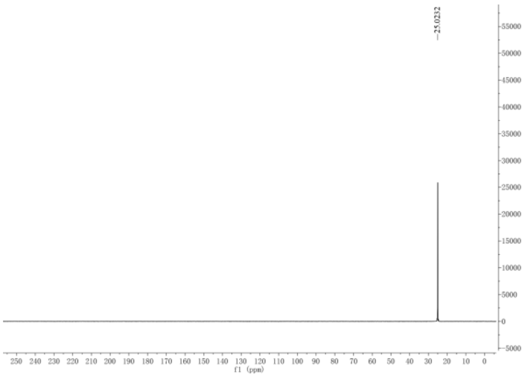

$^{31}P$ NMR of $mitoQNO_6$

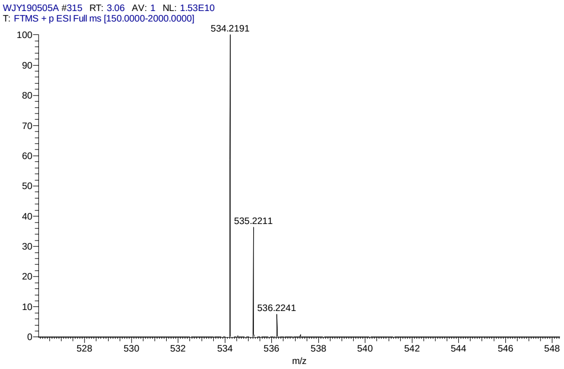


HRMS of $mitoQNO_6$

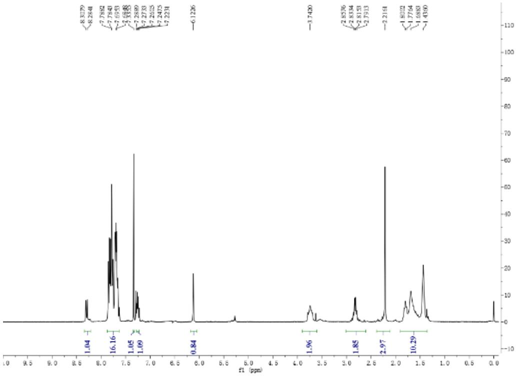

$^1H$ NMR of $mitoQNO_8$

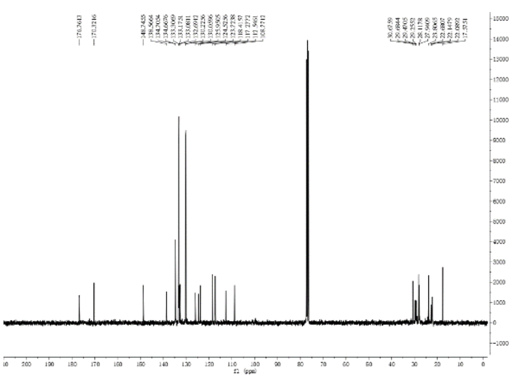

$^{13}C$ NMR of $mitoQNO_8$

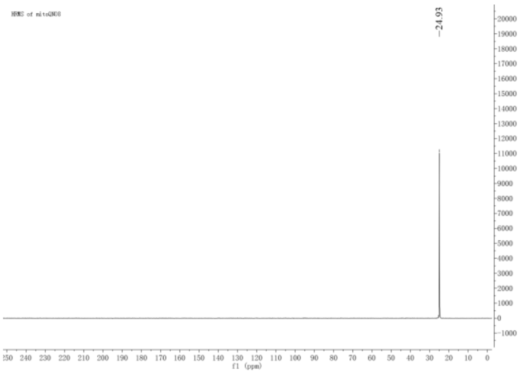


$^{31}$P NMR of mitoQNO$_8$

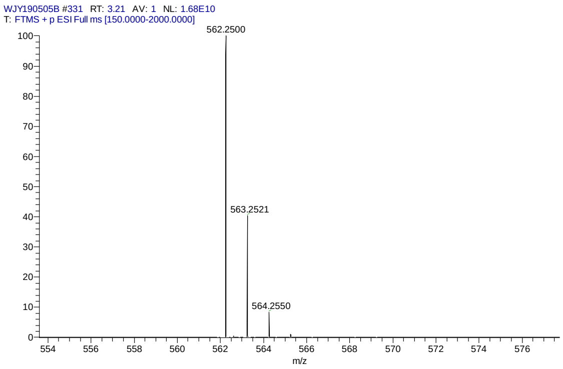


HRMS of mitoQNO$_8$

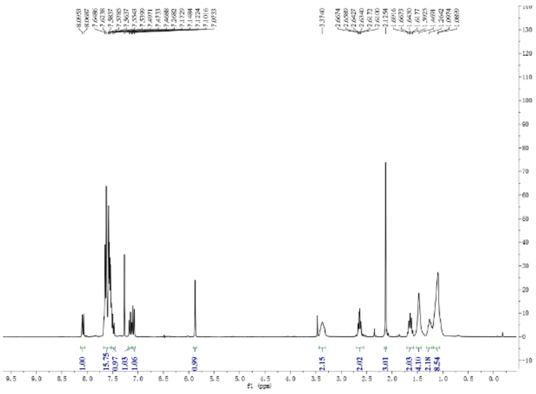


$^{1}$H NMR of mitoQNO$_{11}$

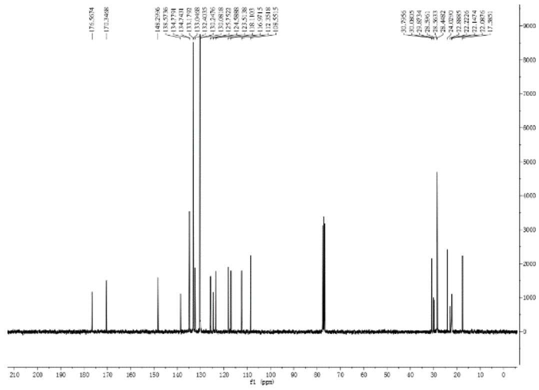


$^{13}$C NMR of mitoQNO$_{11}$

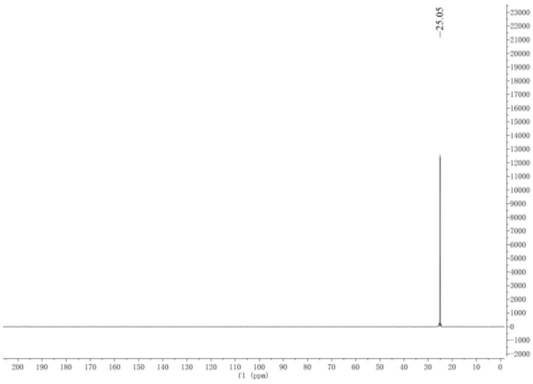


$^{31}$P NMR of mitoQNO$_{11}$

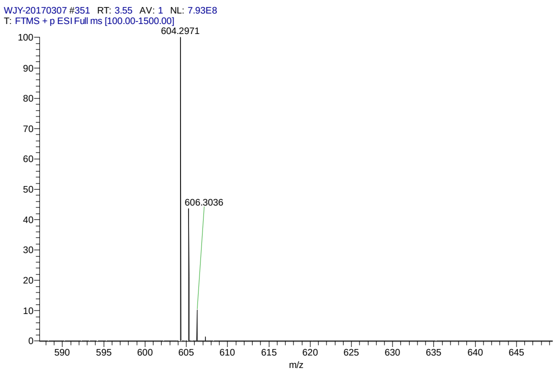


HRMS of mitoQNO$_{11}$
